# Age at menarche and lung function: a Mendelian randomization study

**DOI:** 10.1007/s10654-017-0272-9

**Published:** 2017-06-17

**Authors:** Dipender Gill, Nuala A. Sheehan, Matthias Wielscher, Nick Shrine, Andre F. S. Amaral, John R. Thompson, Raquel Granell, Bénédicte Leynaert, Francisco Gómez Real, Ian P. Hall, Martin D. Tobin, Juha Auvinen, Susan M. Ring, Marjo-Riitta Jarvelin, Louise V. Wain, John Henderson, Deborah Jarvis, Cosetta Minelli

**Affiliations:** 1 Department of Clinical Pharmacology and Therapeutics, Imperial College London, Hammersmith Hospital, London, UK; 2 St. Mary’s Hospital, Imperial College Healthcare NHS Trust, London, UK; 3 Department of Health Sciences, University of Leicester, Leicester, UK; 4 Department of Epidemiology and Biostatistics, School of Public Health, Imperial College London, London, UK; 5 Population Health and Occupational Disease, NHLI, Imperial College London, Emmanuel Kaye Building, 1B Manresa Road, SW3 6LR London, UK; 6 MRC-PHE Centre for Environment and Health, London, UK; 7 School of Social and Community Medicine, University of Bristol, Bristol, UK; 8 UMR 1152, Pathophysiology and Epidemiology of Respiratory Diseases, Epidemiology Team, Inserm, Paris, France; 9 UMR 1152, Univ Paris Diderot - Paris 7, Paris, France; 10 Department of Gynecology and Obstetrics, Haukeland University Hospital, Bergen, Norway; 11 Department of Clinical Science, University of Bergen, Bergen, Norway; 12 Division of Respiratory Medicine, Queen’s Medical Centre, University of Nottingham, Nottingham, UK; 13 National Institute for Health Research, Leicester Respiratory Biomedical Research Unit, Glenfield Hospital, Leicester, UK; 14 Institute of Health Sciences, University of Oulu, Oulu, Finland; 15 Biocenter Oulu, University of Oulu, Oulu, Finland; 16 Center for Life Course Epidemiology, Faculty of Medicine, University of Oulu, Oulu, Finland; 17 Unit of Primary Care, Oulu University Hospital, Oulu, Finland

**Keywords:** Mendelian randomization, Menarche, Puberty, Lung function, FVC, FEV1/FVC

## Abstract

A trend towards earlier menarche in women has been associated with childhood factors (e.g. obesity) and hypothesised environmental exposures (e.g. endocrine disruptors present in household products). Observational evidence has shown detrimental effects of early menarche on various health outcomes including adult lung function, but these might represent spurious associations due to confounding. To address this we used Mendelian randomization where genetic variants are used as proxies for age at menarche, since genetic associations are not affected by classical confounding. We estimated the effects of age at menarche on forced vital capacity (FVC), a proxy for restrictive lung impairment, and ratio of forced expiratory volume in one second to FVC (FEV1/FVC), a measure of airway obstruction, in both adulthood and adolescence. We derived SNP-age at menarche association estimates for 122 variants from a published genome-wide meta-analysis (N = 182,416), with SNP-lung function estimates obtained by meta-analysing three studies of adult women (N = 46,944) and two of adolescent girls (N = 3025). We investigated the impact of departures from the assumption of no pleiotropy through sensitivity analyses. In adult women, in line with previous evidence, we found an effect on restrictive lung impairment with a 24.8 mL increase in FVC per year increase in age at menarche (95% CI 1.8–47.9; *p* = 0.035); evidence was stronger after excluding potential pleiotropic variants (43.6 mL; 17.2–69.9; *p* = 0.001). In adolescent girls we found an opposite effect (−56.5 mL; −108.3 to −4.7; *p* = 0.033), suggesting that the detrimental effect in adulthood may be preceded by a short-term post-pubertal benefit. Our secondary analyses showing results in the same direction in men and boys, in whom age at menarche SNPs have also shown association with sexual development, suggest a role for pubertal timing in general rather than menarche specifically. We found no effect on airway obstruction (FEV1/FVC).

**Electronic supplementary material:**

The online version of this article (doi:10.1007/s10654-017-0272-9) contains supplementary material, which is available to authorized users.

## Introduction

The timing of sexual development in women has shown a secular trend with a shift towards an earlier age over the years [1], and this has been related to childhood life-style and social factors, including diet and obesity, psychological stress and deprivation, as well as environmental exposures, including endocrine disruptors found in many household products [2]. Menarche, defined as the date of the first day of the first menstrual bleeding, is preceded by a complex hormonal cascade and signals the initiation of the menstrual cycle in adolescent girls. Earlier age at menarche has been described as a risk factor for a number of adverse health outcomes, including obesity [3], type 2 diabetes [4], cardio-metabolic traits [5], cardiovascular morbidity and mortality [6], as well as breast [7] and ovarian [8] cancers. Understanding the effects of the age at menarche offers insight into the pathophysiology of related diseases. In particular this can highlight the potential effects of early and late exposure to sex hormones on health outcomes in women, as well as help explain gender differences in the risks of common diseases [9–11].

Timing of menarche has been considered an important factor in relation to respiratory health [12]. Lung function is an important predictor of both respiratory disease and overall health. After cessation of lung growth by the early twenties, there is a plateau in lung function followed by gradual age-related decline. There are two common patterns of lung function impairment, obstruction and restriction, and these have a different impact on morbidity and mortality [13]. Obstruction, measured as a low ratio of the forced expiratory volume in one second (FEV_1_) to the forced vital capacity (FVC), represents an objective marker of chronic obstructive pulmonary disease, a major and growing cause of morbidity, disability and death worldwide. Restriction, defined as low total lung capacity (measured by plethysmography) but commonly approximated by low FVC in population-based studies, is a predictor of all-cause mortality even in the absence of chronic respiratory conditions. There has been increasing interest in understanding gender-related risk factors for lung function impairment, particularly in relation to hormonal influences and sexual development. Substantial evidence shows that lung function is influenced by sex hormones in women [14] for whom low lung function has been associated with irregular menstruation, menopausal transition, and both natural and surgical menopause, with report of improvement in lung function in postmenopausal women receiving hormone replacement therapy [12, 15].

An observational study of 2873 women aged 27–57 years investigated the association of early menarche with adult lung function and found a lower FVC and FEV_1_, but not FEV_1_/FVC, in women with early menarche [16]. This work took account of important potential confounders, including age, height, body mass index (BMI), smoking, education and birth order, as well as secular trends (age at menarche has changed over the years and lung function has also changed due to changes in height). However, the effect of residual confounding by unmeasured, or poorly recalled, early life and childhood factors cannot be ruled out. For example, age at menarche is influenced by childhood nutritional status [17], which in turn might influence lung function in adult life; another example is birth weight, which has been associated with both age at menarche [18] and lung function [19]. Such limitation is typical of observational studies, where confounding can make it hard to distinguish between causal effects and spurious associations.

Mendelian randomization (MR) can help assess the causality of an observed association by using genetic variants as proxies, or “instrumental variables”, for the exposure of interest [20, 21]. Genetic associations are not typically affected by confounding or reverse causation because genes are randomly allocated at the time of conception. Provided the underlying assumptions are satisfied [22], the demonstration that genetic variants known to modify age at menarche also modify lung function provides indirect evidence of a causal effect of age at menarche. The MR technique has been rapidly growing in popularity because of these advantages over the classical epidemiological approach, with MR studies having previously investigated age at menarche as a risk factor for depression [23], and lung function as an outcome in relation to C-reactive protein [24].

In this study we used MR to investigate the lifetime effect of age at menarche on lung function in adolescent girls and adult women, using 122 single nucleotide polymorphisms (SNPs) associated with age at menarche. We considered the effects in both adulthood and adolescence, since we hypothesised that they could differ due to a different role of menarche in lung growth, maximal lung function attained, and lung function decline.

## Methods

### SNP-age at menarche association estimates

We derived SNP-age at menarche association estimates from a published genome-wide association (GWA) meta-analysis of 57 studies on 182,416 women of European descent, which identified 122 independent SNPs at 106 genomic loci (*p* value < 5 × 10^−8^) [25]. Overall, the 122 SNPs explained 2.7% of the variability of age at menarche in the population. Age at menarche was based on self-reporting and analysed as a continuous variable, with study-specific analyses adjusted for birth year, to account for the secular trends in menarche timing, and genomic control, to account for population stratification [25]. We assessed instrument strength for the 122 SNPs, a function of magnitude and precision of their genetic effect, using the F statistic [26].

### SNP-lung function association estimates

Three studies were used to estimate the association of the 122 SNPs with lung function in adult women: European Community Respiratory Health Survey (ECRHS) [27], Northern Finland Birth Cohort of 1966 (NFBC 1966) [28], and UK Biobank [29] (Table 1). ECRHS is a European prospective cohort study designed to identify risk factors for respiratory health [27]. The study started in 1992 (ECRHS I), with follow-up performed twice (ECRHS II and III) over the following 20 years. Here we include 1069 women aged 27–57 recruited at random from population-based sampling frames in 14 centres, and who had lung function measured in ECRHS II. Genetic associations analyses for the 122 SNPs with FVC and FEV_1_/FVC were adjusted for age, age^2^, height, centre and ancestry principal components. NFBC1966 is a birth-cohort study performed in the Finnish provinces of Oulu and Lapland. Pregnant women with expected date of delivery in 1966 were recruited and their offspring followed up [28]. Among the offspring, we include 2680 women with spirometry data at age 31. Analyses were adjusted for height and ancestry principal components. UK Biobank is a prospective study across 22 assessment centres, aimed at identifying causes of chronic disease in middle and old age [29]. We include 43,195 women of European ancestry aged 40–69 recruited in 2006–2010, who had GWA and lung function data. Analyses were adjusted for age, age^2^, height, and ancestry principal components, as well as smoking pack-years because part of the sample was ascertained by smoking status [30].

**Table 1:** Characteristics of the study populations included for the SNP-lung function associations

Study	Study design	Sample size (N)	Age at spirometry (years)	Age at menarche (years)	FVC (ml)	FEV_1_ (ml)	FEV_1_/FVC (%)
*Adult women*							
ECRHS II	Multicentre cohort	1069	42.9 (7.1)	12.9 (1.5)	3677 (617)	2927 (531)	80 (7.2)
NFBC 1966	Birth cohort	2680	31 (0)	12.9 (1.3)	4014 (552)	3407 (461)	85 (6.3)
UK Biobank	Multicentre cross-sectional	43,195	56.6 (7.7)	12.9 (1.6)	3094 (675)	2251 (574)	75 (6.8)
*Adolescent girls*							
ALSPAC	Birth cohort	1234	15.5 (0.3)	12.5 (1.3)	3294 (599)	3005 (545)	92 (6.8)
NFBC 1986	Birth cohort	1791	16 (0)	12.5 (1.1)	3758 (497)	3353 (446)	89 (7.0)

Values reported are mean (standard deviation)

We used two studies to estimate the association of the 122 SNPs with lung function in adolescent girls: Avon Longitudinal Study of Parents and Children (ALSPAC) [31] and Northern Finland Birth Cohort of 1986 (NFBC 1986) [32] (Table 1). ALSPAC is a birth-cohort study that initially enrolled 14,541 pregnant women in Bristol, United Kingdom, in 1990–1992 [31]. We include 1234 of their daughters, aged around 16, with GWA and spirometry data. Analyses were adjusted for height; ancestry principal components were not included because there was no evidence of population stratification in the study. NFBC 1986 is a Finnish birth-cohort study that followed up 9432 live births to mothers in Oulu and Lapland. A total of 6642 adolescents aged 16 participated in the clinical examination in 2001–2002, and here we include 1791 with available GWA and lung function data [32]. Analyses were adjusted for height and ancestry principal components. For both ALSPAC and NFBC 1986, robust standard errors were used for the analyses of FEV_1_/FVC to account for deviation from normality of the regression residuals.

Spirometry methods for all studies are reported in Supplementary Table 1. For adults and adolescents, estimates of the association with FVC and FEV_1_/FVC for each SNP were pooled across studies using fixed-effect inverse-variance weighted meta-analysis.

### Mendelian randomization estimates

We used a two-sample MR approach for summary data with multiple instruments, where the estimate of the causal effect is obtained as the inverse-variance weighted combination of individual MR estimates across instruments, using fixed-effect meta-analysis [33]. Individual MR estimates for the 122 SNPs were derived using the Wald estimator (ratio of SNP-lung function estimate over SNP-age at menarche estimate), with standard error derived using the delta method [34].

### Investigation of pleiotropy

A fundamental assumption of MR is the absence of pleiotropy, i.e. the genetic instruments modify lung function only through age at menarche and no other independent pathways [22]. We tested for statistical evidence of pleiotropy by using between-instrument heterogeneity as a proxy; in the absence of pleiotropy, all variants are valid instruments and their MR estimates will vary only by chance (no heterogeneity) [35]. We defined evidence of pleiotropy as an I^2^ > 25%, where I^2^ describes the percentage of total variation in MR estimates due to heterogeneity rather than chance [35], or a statistically significant heterogeneity Cochran Q test (*p* < 0.05). If pleiotropy was detected, we performed a series of sensitivity analyses to address it.

Using the PhenoScanner, a curated database of publicly available GWA findings created to inform MR studies (available at www.phenoscanner.medschl.cam.ac.uk/phenoscanner) [36], we first checked for previous associations of the 122 SNPs (and highly correlated SNPs; linkage disequilibrium r^2^ > 0.8) with *any* phenotype other than age at menarche, limiting our search to associations that had been identified at a significance level of 5 × 10^−8^ (Supplementary Table 2). For the exclusion of possible pleiotropic SNPs in our sensitivity analyses, we then only considered effects on phenotypes which can be related to lung function. We adjusted for height all SNP-lung function analyses and therefore height could not directly induce pleiotropy. However, height SNPs could be associated with other markers of somatic growth, potentially exerting pleiotropic effects on lung function other than through height. To test this, we excluded SNPs previously associated with height. We then additionally excluded SNPs previously associated with obesity and related traits (weight, BMI, waist circumference), fasting insulin and type 2 diabetes, and birth weight, since these might also influence lung function. These exclusions were used in sensitivity analyses if there was statistical evidence of pleiotropy.

If statistically significant heterogeneity remained after the exclusion of these SNPs, we performed two further sensitivity analyses: a meta-analysis of MR estimates using a random-effects model instead of the fixed-effect model used in the main analysis [37], and MR-Egger regression [38]. The random-effects model allows for random pleiotropic effects across SNPs, while MR-Egger regression provides unbiased results if pleiotropic effects are not random (i.e. do not cancel). Both analyses assume that the magnitude of the pleiotropic effects is independent of the magnitude of the corresponding SNP-age at menarche effects. As these approaches are less powerful than the fixed-effect meta-analysis, particularly the MR-Egger regression [37], they were only used as further sensitivity analyses when between-instrument heterogeneity was still present after excluding possible pleiotropic SNPs.

In order to understand whether the observed effects of age at menarche on FVC were due to factors specific to menarche rather than puberty in general, we tested the association of our 122 SNPs with lung function in men. Many of the age at menarche SNPs that we used as instruments have also been shown to regulate male pubertal timing as measured by Tanner stage [25]. Finding evidence of an association in men would indicate that the underlying mechanism is related to general timing of puberty as opposed to a female-specific effect. SNP–FVC associations in adolescent boys (N = 3421) and adult men (N = 40,687) were estimated from the same studies used for women (Supplementary Table 6). For each SNP, association estimates were pooled across studies using fixed-effect inverse-variance weighted meta-analysis. The individual SNP–FVC estimates were then meta-analysed (fixed-effect model) to provide an overall effect of the 122 SNPs on FVC, which is equivalent to performing an unweighted allele score analysis with all SNPs.

All analyses were performed using Stata 14 (StataCorp LP)

## Results

Imputed genotype data for the 122 SNPs were available for all studies, with the exception of one SNP (rs10423674) in NFBC 1986. The quality of imputation was very good for all SNPs across all studies (imputation INFO or R^2^ parameters ≥ 0.8), except for one SNP in two studies (rs17233066, R^2^ of 0.4 in ECRHS II and ALSPAC). The SNPs identified were strong instruments for age of menarche. F statistics ranged from 21 to 441 across variants (Supplementary Table 3), well over the threshold of F > 10 usually recommended as a test for weak instruments in MR analyses [39].

Individual estimates of the per-allele effects on age at menarche and lung function (FVC and FEV_1_/FVC) for each SNP are provided in Supplementary Tables 3 and 4, respectively. MR estimates for the causal effect of age at menarche on lung function obtained separately from each SNP are presented in Supplementary Table 5, while the combined MR estimates across the 122 SNPs are reported in Table 2.

**Table 2:** MR estimates for the causal effect of age at menarche on lung function in adults and adolescents, obtained by fixed-effect meta-analysis of SNP-specific MR estimates across the 122 SNPs

Population	Sample size	MR estimate		Between-instrument heterogeneity	
		Beta (95% CI)	*p* value	I^2^ (95% CI)	Het. *p* value
*FVC*					
Adult women	46,944	24.8 (1.8 to 47.9)	**0.035**	45 (31 to 55)	<0.001
Adolescent girls	3025	−56.5 (−108.3 to −4.7)	**0.033**	2 (0 to 21)	0.418
*FEV*_*1*_ */FVC*					
Adult women	46,944	0.0 (−0.7 to 0.7)	0.968	0 (0 to 19)	0.618
Adolescent girls	3025	0.6 (−0.2 to 1.4)	0.157	3 (0 to 23)	0.386

Beta, estimate of effect of 1 year increase in age at menarche on FVC (mL) and FEV_1_/FVC (%); I^2^ (%), between-instrument heterogeneity; Het. *p* value, Q test *p* value

Bold values indicate statistically significant *p* values

The MR estimate for age at menarche and FVC in adult women showed a statistically significant increase of 24.8 mL per year increase in age at menarche (95% confidence interval 1.8–47.9; *p* = 0.035), while we found no effect for FEV_1_/FVC (Table 2). In the MR analysis for FVC, a between-instrument I^2^ of 45% (95% CI 31–55%; *p* < 0.001) suggested the presence of pleiotropy, and we repeated the analysis after excluding SNPs with potentially pleiotropic effects. Out of the 122 SNPs, 34 had been previously associated with phenotypes other than age at menarche, the large majority of which were height and obesity-related traits (Supplementary Table 2). The first sensitivity analysis excluding 14 SNPs associated with height (Model 1 in Table 3) showed a larger and more highly statistically significant MR estimate (43.6 mL; 17.2–69.9; *p* = 0.001). The second sensitivity analysis, where we additionally excluded 13 SNPs associated with other traits potentially related to lung function, showed very similar results (Model 2 in Table 3). Since some residual between-instrument heterogeneity remained in both sensitivity analyses (Table 3), we performed a random-effects meta-analysis of the MR estimates as well as MR-Egger regression. For the random-effects meta-analysis, results were similar to those in Table 3, with an MR estimate of 40.7 mL (8.4–72.9; *p* = 0.013) for Model 1, and 40.3 mL (5.7–74.8; *p* = 0.022) for Model 2. MR-Egger regression, which suffers from low statistical power [37], showed results in the same direction but with much larger confidence intervals and loss of statistical significance (Model 1: 95.7 mL, −37.0 to 228.4, *p* = 0.156; Model 2: 84.8 mL, −52.2 to 221.8, *p* = 0.222).

**Table 3:** Sensitivity analyses for the MR of age at menarche and FVC in adult women

Model	Number of SNPs	MR estimate		Between-instrument heterogeneity	
		Beta (95% CI)	*p* value	I^2^ (95% CI)	*Het. p* value
*Model 1* Excluding SNPs previously associated with height	108	43.6 (17.2–69.9)	**0.001**	31 (12–46)	0.002
*Model 2* Excluding SNPs previously associated with height, obesity, weight, BMI, waist circumference, fasting insulin, type 2 diabetes, or birth weight	95	42.9 (14.7–71.2)	**0.003**	31 (10–46)	0.003

Reported are MR estimates after excluding SNPs with possible pleiotropic effects (Suppl. Table 2). Beta, estimate of effect of 1 year increase in age at menarche on FVC (mL); I^2^ (%), between-instrument heterogeneity; Het. *p* value, Q test *p* value

Bold values indicate statistically significant *p* values

In adolescents, the MR analysis for FVC showed a statistically significant *decrease* of 56.5 mL per year increase in age at menarche (95% CI −108.3 to −4.7; *p* = 0.033), with no evidence of pleiotropy across the 122 SNPs (Table 2). As with adults, there appeared to be no causal effect of age at menarche on FEV_1_/FVC.

The results of the secondary analyses in men were very consistent with those in women, with a statistically significant positive association of the 122 SNPs with FVC in adult men (*p* = 0.013) and a statistically significant negative association in adolescent boys (*p* = 0.007).

## Discussion

Our study shows a causal effect of age at menarche on lung function using Mendelian randomization, a technique which draws on the biological principle that genes are randomly allocated at conception to provide evidence not affected by classical confounding. We found an effect of age at menarche on restrictive lung impairment (FVC), with no evidence of an effect on airway obstruction (FEV_1_/FVC). In particular, we find that early menarche increases FVC in adolescence but decreases it in adulthood. The findings for adult women confirm previous observational evidence suggesting a decrease in FVC of 123 mL (95% CI 27–220; *p* = 0.01) associated with early menarche (menarche ≤10 years vs. menarche at 13), but no association with FEV_1_/FVC (*p* = 0.77) [16].

**Fig. 1:**
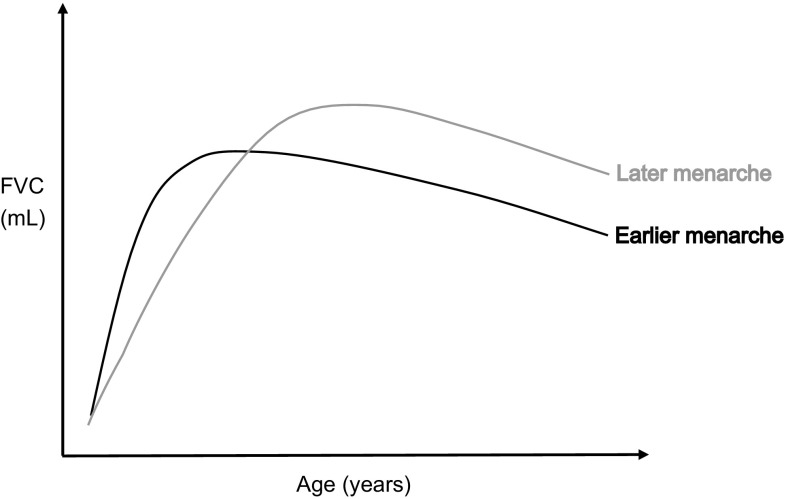
Graphical representation of a possible explanation for the discrepancy in FVC findings for adult women and adolescent girls. Earlier menarche may have current benefits to the lung function in adolescents, but may also lead to premature completion of lung development with attainment of a lower maximal lung function in adult life

The finding of a beneficial effect of earlier age at menarche on FVC in adolescence, as opposed to the detrimental effect in adulthood, is interesting and has a plausible explanation, illustrated graphically in Fig. 1. Lung development tends to plateau following menarche [40], and therefore earlier initiation of menstruation may lead to premature completion of lung development and lower maximally attained lung function. Given the relative stability of lung function over time (a phenomenon known as “tracking”) [41], this would translate to lower FVC in adulthood. Our secondary analysis suggests that the same happens in males, where lung development has also been shown to plateau at puberty [40]. The beneficial effect of earlier menarche on FVC in adolescent girls may be explained by the prominent truncal (as opposed to limb) growth and increased thoracic muscle strength which occur in puberty and which contribute to higher lung volumes [40]. It could also be related to the direct effect of early exposure to sex hormones, for example oestrogens, in adolescent girls, as these have been shown to affect lung function in humans [12] and animal models [42]. Our secondary analysis suggesting a similar effect in boys support the hypothesis of a mechanism related to factors associated with early pubertal timing in general rather than specifically through female sex hormones. However, it is also possible that the mechanisms differ in men and women, for example through sex hormones in girls and thoracic growth and muscle strength in boys (the latter being more pronounced in boys [40]). The complex hormonal and physiological shifts that occur in women during menarche [12] make it difficult to pin-point the precise mechanisms underlying our findings, and further research is needed to explore them.

Our study suggests that if these same adolescents were assessed in early adult life (once they have reached maximal lung function), those with early menarche would have comparatively lower FVC. Large-scale studies of lung function with longitudinal data across the lifespan will allow to test this hypothesis. To date few child cohorts have lung function assessments in adolescence and at ages associated with lung function plateau, but ongoing consortium-based initiatives, such as STELAR [43] and MEDALL [44], will be able to provide the relevant information.

Our findings offer insight into plausible pathophysiological mechanisms underlying the effects of early menarche on lung function impairment. Previous work has shown an association of early menarche with greater risk and severity of asthma [16, 45, 46], while we did not find an effect of age at menarche on airway obstruction in either adults or adolescents. This might be explained by an association of early menarche with bronchial hyperactivity through immunological and inflammatory effects [12, 47], which would manifest as short-term reversible airway obstruction not captured by the FEV_1_/FVC ratio from single assessments in population-based studies. Our study also highlights the importance of evaluating different lung function parameters. Many epidemiological studies on lung function have focused on low FEV_1_, which may arise from either obstructive or restrictive lung impariment. We found that early menarche only affects FVC, a proxy for total lung capacity and characteristic of restrictive lung impariment, which is a predictor of morbidity (including cardiovascular morbidity) and mortality even in the absence of chronic respiratory conditions [13].

All 122 SNPs used in our MR study were “strong” instruments, with strength reflecting not only the magnitude of the genetic effects on age at menarche but also the precision of their estimates. This is important since the use of “weak” instruments can bias the MR estimate [39], with such bias resulting in an attenuation of the causal effect in the context of a two-sample MR analysis like ours [48]. To improve precision, we used the results from the GWA discovery rather than replication analysis from Perry et al. [25], as the former was over 20 times larger (182,416 vs. 8689). These estimates might have been affected by the upward bias typical of the discovery stage (“winner’s curse”) [49], but this is likely to be very limited in our study given the strong *p* values. Moreover, any resulting overestimation of the SNP-age at menarche association would have pulled the MR estimate towards the null, leading to underestimation of the true causal effect rather than to a false positive result.

Like any other instrumental variable approach, MR tends to suffer from limited statistical power when the effect of the instruments on the exposure is relatively small, as typically happens with common genetic variants [50]. Despite this, we were able to identify statistically significant effects of age at menarche on FVC, although the confidence intervals of our MR estimates are large, particularly for adolescents where the sample size for the genetic associations with lung function is much smaller.

MR is not affected by classical confounding encountered in observational studies, and yet there is a form of confounding specific to MR, pleiotropy, whereby the genetic instrument modifies lung function through secondary phenotypes other than age at menarche [22]. Heterogeneity in the MR estimates obtained from the individual instruments can be used as a proxy for pleiotropy [35]. In our study there was evidence of heterogeneity in the MR estimates for the analysis of FVC in adults; the exclusion of SNPs with possible pleiotropic effects, in particular SNPs previously associated with height, showed consistent and much stronger results than the main analysis, thus demonstrating robustness of our findings. Height is strongly influenced by genetic and environmental factors regulating growth and development, and is also a strong predictor of FVC. In order to clearly disentangle the effect of the age at menarche SNPs on FVC from any possible effect on height, we adjusted all our SNP-lung function analyses for height. Indeed, it is FVC standardised for height which is clinically of interest, and FVC is often expressed as a percentage of a normal reference value (percent predicted) based on the individual’s height as well as sex and age. The fact that removal of SNPs previously associated with height reduced the pleiotropy and made the result stronger in our secondary analysis in adult women supports our hypothesis that these SNPs could be associated with other markers of somatic growth, exerting pleiotropic effects on lung function other than through height. Robustness of our finding for FVC in women was also confirmed by a further analysis to account for residual pleiotropic effects using a random-effects meta-analysis, while MR-Egger regression resulted in a loss of statistical significance likely explained by its low statistical power [37]. Interestingly, there was no statistical evidence of pleiotropy for FVC in adolescents, as shown by an I^2^ of 2% for the between-instrument heterogeneity (95% CI 0–21%; *p* = 0.42). A possible explanation for this is that some of our genetic instruments may have effects on secondary phenotypes related to lung function that only become apparent during adult life.

A potential weakness of our study may arise from the presence of gene-environment interactions [51], since our MR analyses assume no interactions for the SNP-age at menarche and SNP-lung function relationships. For example, the data used in our study for the analysis of adults and adolescents cover different “cohorts” of women, potentially exposed to different environmental exposures. “Cohort effects” are known to affect both age at menarche and lung function. The MR approach is not susceptible to confounding from environmental exposures, including those inducing cohort effects, but they might bias the results if they interacted with the genetic variants used as instrumental variables [52]. Although the practical relevance of gene-environment interactions for our MR analyses of age at menarche and lung function is not clear, it remains a theoretical possibility.

Finally, our MR estimate of the effect of age at menarche on FVC needs to be interpreted as a *population*-*averaged* causal effect rather than the effect for an individual and are based on the assumption of a linear relationship. Parametric and non-parametric methods to address non-linearity in MR have been proposed, including stratification of the exposure to estimate localized average causal effects (LACE) [53, 54], although they typically require individual-level data.

In conclusion, our study provides evidence of a causal effect of early sexual development in women on lung function later in life, with our secondary findings in men suggesting a role for pubertal timing in general rather than menarche specifically. This, together with evidence of detrimental effects on other adverse health outcomes, including cardiometabolic outcomes and cancer [3–8], has public health implications given that factors predisposing to early sexual development in women could be targeted at a population level to contrast the secular trend towards earlier puberty. These include a number of established childhood life-style and social factors, such as diet and obesity, psychological stress and deprivation, as well as hypothesised environmental exposures, such as endocrine disrupting chemicals (EDCs) found in many household products [2]. Of these, childhood obesity is the most worrisome given the current childhood obesity epidemic. EDCs may prove to be a substantial concern due to their widespread presence and the potential persistence of their effects on menarche for generations without further exposure (transgenerational inherited effects) [55], although these effects remain controversial. Our study also illustrates the value of the MR approach, which exploits increasingly available genetic data from large datasets, as a tool to investigate causal effects of childhood events on adult health, an area of epidemiological research which is particularly problematic due to the presence of confounding factors very difficult to measure and partly unknown.

## Electronic supplementary material

Below is the link to the electronic supplementary material.

Supplementary Table 1 Spirometry methods for all studies on lung function included (PDF 186 kb)

Supplementary Table 2 Evidence of association of the 122 SNPs (and SNPs highly correlated with them, LD r2 > 0.8) with secondary phenotypes. Information retrieved from the PhenoScanner (36) (available at: www.phenoscanner.medschl.cam.ac.uk/phenoscanner) (PDF 325 kb)

Supplementary Table 3 Estimates of the SNP-age at menarche association (GX) for all 122 SNPs, from Perry et al. EA: effect allele; EAF: effect allele frequency; R^2^: proportion of the variance of age at menarche explained by the SNP, calculated as follows: R^2^ = [2 × EAF × (1–EAF) × β^2^]/varX, where β is the estimated genetic effect on age at menarche and varX is the variance of age at menarche; F: F statistic, a function of the magnitude and precision of the genetic effect calculated as: F = R^2^(N − 2)/(1 − R^2^), where N is the sample size of the SNP-age of menarche association (N = 182, 416); GX: per-allele genetic effect on age at menarche (years); GX SE: standard error of GX; p: p value of GX (PDF 401 kb)

Supplementary Table 4 Estimates of the SNP-lung function association (GY) for all 122 SNPs, for adult women (ECRHS, NFBC 1966 and UK Biobank studies) and adolescent girls (ALSPAC and NFBC 1986 studies). For one SNP, rs10423674, data were missing from NFBC 1986. EA: effect allele; GY: per-allele genetic effect on FVC (ml) or FEV_1_/FVC (%); GY SE: standard error of GY (PDF 407 kb)

Supplementary Table 5 Estimates of the causal effect of age at menarche on lung function for all 122 SNPs, for adult women (ECRHS, NFBC 1966 and UK Biobank studies) and adolescent girls (ALSPAC and NFBC 1986 studies). EA: effect allele; Beta: estimate of the effect of one year increase in age at menarche on FVC (ml) or FEV_1_/FVC (%); Beta SE: standard error of beta (PDF 499 kb)

Supplementary Table 6 Characteristics of the study populations included for the SNP-lung function associations in adult men and adolescent boys. Values reported are mean (standard deviation) (PDF 312 kb)
