# Supplementary material for: Age at menarche and lung function: a Mendelian randomization study
Source: Eur J Epidemiol. 2017 Jun 17;32(8):701–10. doi: 10.1007/s10654-017-0272-9 (PMC5591357; doi:10.1007/s10654-017-0272-9)
Supplement: Supplementary file 1 — Spirometry methods for all studies on lung function included (PDF 186 kb) [file 10654_2017_272_MOESM1_ESM.pdf]

Supplementary Table 1. Spirometry methods for all studies on lung function included. ATS: American Thoracic Society

Study	Spirometer	Methods
ECRHS II	Biomedin water-sealed spirometer (Biomedin, Padova, Italy): 9 centres<br><br>SensorMedics spirometer (Sensormedics, Yorba Linda, USA): 4 centres<br><br>Jaeger Pneumo Lab spirometer (Jaeger, Würzburg, Germany): 3 centres	- Spirometric manoeuvres performed in the sitting position with nose clips; up to nine attempts performed to provide at least two technically acceptable manoeuvres<br><br>- Volume signal of equipment verified on a daily basis using calibrated syringes (2 or 3 L)<br><br>- Spirometry data included only if forced expiratory manoeuvre satisfactory and compliant with ATS criteria
NFBC1966	Vitalograph P-model spirometer (Vitalograph Ltd., Buckingham, UK)	- Spirometric manoeuvres performed three times, but repeated if coefficient of variation between two maximal readings >4%<br><br>- Spirometer calibrated regularly, with a volumetric accuracy of ±2% or ±50 mL, whichever was greater
UK Biobank	Vitalograph Pneumotrac 6800, Buckingham, UK	- The participant was asked to record two to three blows (lasting for at least 6 seconds) within a period of about 6 minutes. The reproducibility of the first two blows was compared and, if acceptable (defined as a <5% difference in FVC and FEV_1), a third blow was not required.<br><br>- Spirometry data included if ≥2 measures passing ATS/ERS criteria (1).
ALSPAC	Vitalograph 2150 Spirometer ((Vitalograph Ltd., Buckingham, UK)	- Spirometric manoeuvres performed in upright sitting position with nose clip<br><br>- Spirometric manoeuvres performed up to eight times to achieve three technically acceptably, reproducible blows (ATS/ERS criteria) with FVC within ±150mL<br><br>- Spirometer calibrated daily with 3L syringe to volumetric accuracy ±3%.
NFBC1986	Vitalograph 2150 spirometer (Vitalograph Ltd., Buckingham, UK)	- Spirometric manoeuvres performed in a upright sitting position with nose clips<br><br>- Spirometric manoeuvres performed three times, but repeated if coefficient of variation between two maximal readings >4%

1. Miller MR, Hankinson J, Brusasco V, Burgos F, Casaburi R, Coates A, et al. Standardisation of spirometry. Eur Respir J. 2005;26(2):319-38.
