# Supplementary material for: Age at menarche and lung function: a Mendelian randomization study
Source: Eur J Epidemiol. 2017 Jun 17;32(8):701–10. doi: 10.1007/s10654-017-0272-9 (PMC5591357; doi:10.1007/s10654-017-0272-9)
Supplement: Supplementary file 2 — Evidence of association of the 122 SNPs (and SNPs highly correlated with them, LD r2 > 0.8) with secondary phenotypes. Information retrieved from the PhenoScanner (36) (available at: www.phenoscanner.medschl.cam.ac.uk/phenoscanner) (PDF 325 kb) [file 10654_2017_272_MOESM2_ESM.pdf]

Supplementary Table 2. Evidence of association ($p<5x10^{-8}$) of the 122 SNPs and SNPs highly correlated with them (LD r2>0.8) with secondary phenotypes. Information retrieved using the PhenoScanner, available at: www.phenoscanner.medschl.cam.ac.uk/phenoscanner (1). Chr: chromosome.

SNP	Chr: Position	Gene	Secondary phenotypes
rs10144321	14:100416068	WDR25	/
rs1038903	4:28750432		/
rs10423674	19:18707093	CRTC1	/
rs10453225	9:106157939		Height (2)
rs10739221	9:106298549		/
rs10789181	1:65350884	DNAJC6	/
rs1079866	7:41430495		/
rs10816359	9:105995389		/
rs10895140	11:101565990	TRPC6	/
rs10938397	4:45180510		Body Mass Index (3-9)<br>Hip circumference (10)<br>Obesity (8)<br>Waist circumference (5, 10)<br>Weight (5)
rs10980854	9:111288077		/
rs10980921	9:111517632		Height (2)
rs11022756	11:13293892	ARNTL	/
rs11165924	1:97909892	DPYD	/
rs11215400	11:115181915	CADM1	/
rs1129700	16:29906713	ASPHD1, KCTD13	/
rs11578152	1:102111465		/
rs11715566	3:117843589		/
rs11767400	7:122520688	CADPS2	/
rs11792861	9:109047015	MIR32, TMEM245	/
rs12148769	15:23906947		/
rs12446632	16:19924067		BMI (4-8)<br>Hip circumference (10)<br>Obesity (8)<br>Waist circumference (10)<br>Weight (5, 8)
rs12472911	2:141470940	LRP1B	/
rs1254337	14:60453807	C14orf39	Height (2, 5, 8, 11)
rs12571664	10:119949417		/
rs12607903	18:3817134	DLGAP1	/
rs12915845	15:88499236		/
rs13053505	22:38849613		/
rs13067731	3:137271149		Height (2)
rs13135934	4:94286537	SMARCAD1	/
rs13179411	5:134564823	JADE2	/
rs13196561	6:100312294		/
rs1324913	13:74061451	KLF12	/
rs1364063	16:69554669		Height (2)
rs1400974	2:198773966		/
rs1461503	11:122974367		Height (2)
rs1469039	8:139639536	KCNK9	/
rs1532331	5:43116728		/
rs16860328	3:185917897	TRA2B	Height (2)

SNP	Chr: Position	Gene	Secondary phenotypes
rs16896742	6:29954963		Rheumatoid arthritis (12)
rs16918254	8:52856653		/
rs16918636	11:29102635		/
rs17086188	5:96510150		/
rs17171818	5:138389314	KDM3B	/
rs17233066	2:198779314		/
rs17236969	2:155895947		/
rs17266097	2:199410486	SATB2	/
rs1874984	10:1689677	ADARB2	/
rs1915146	10:125157645	CTBP2	/
rs1958560	14:65570077	FUT8	Glycosylation of immunoglobulin G (13)
rs2063730	11:78337478	GAB2	/
rs2137289	18:47225754	SKOR2	/
rs2153127	6:104900669		Height (2, 5)
rs2274465	1:43655886	KDM4A	/
rs239198	6:100686201	ASCC3	/
rs244293	17:55153361	STXBP4	/
rs246185	16:14301575	MIR193BHG	Height (2, 11, 14-16)<br>Puberty onset genital enlargement (17)<br>QT interval (18)
rs2479724	6:41923244	BYSL	Height (2)<br>Mean corpuscular haemoglobin (19)<br>Mean corpuscular volume (19)
rs251130	5:111524099	STARD4-AS1	/
rs2600959	3:132896620		/
rs268067	2:59653910		/
rs2687729	3:128176383	EEFSEC	Hypospadias (20)
rs2688325	8:3909688	CSMD1	/
rs2836950	21:39232503	BRWD1	/
rs2947411	2:614168		Body fat percentage (21)<br>BMI (4-8)<br>Hip circumference (10)<br>Obesity (8, 22)<br>Overweight (5, 8)<br>Waist circumference (5, 10)
rs3101336	1:72285502		BMI (4-8, 23, 24)<br>Hip circumference (10)<br>Obesity (8, 24)<br>Overweight (5, 8, 23)<br>Waist circumference (10)
rs3733631	4:103719946	TACR3	/
rs3743266	15:60489314	RORA-AS1,<br>RORA	/
rs4369815	2:156270452		/
rs466639	1:165425645	RXRG	/
rs4756059	11:46129068		/
rs4840086	6:99760562		/
rs4875053	8:143790241		/
rs4895808	6:126460288	CENPW	Height (2, 5, 8, 11)
rs4929947	11:8618447	TRIM66	BMI (6, 7)<br>Obesity (8)<br>Waist circumference (10)
rs543874	1:177920345		Body fat percentage (21)<br>BMI (4-9, 25, 26)

SNP	Chr: Position	Gene	Secondary phenotypes
			Hip circumference (10)<br>Obesity (4, 8, 24)<br>Overweight (4, 5, 8)<br>Waist circumference (5, 10)
rs6009583	22:49281720		/
rs6427782	1:199829211		/
rs652260	19:7835676	EVI5L	/
rs6555855	5:169322733		/
rs6563739	13:39665648	COG6,<br>MIR4305	Rheumatoid arthritis (27)
rs6747380	2:56360614	CCDC85A	/
rs6758290	2:105248369		/
rs6762477	3:50055776	RBM6	Ulcerative colitis (28)
rs6770162	3:24669522		/
rs6933660	6:151482619		/
rs6938574	6:128069835	PTPRK	/
rs6964833	7:74687575	GTF2I	/
rs7037266	9:6942940	KDM4C	/
rs7103411	11:27678578	BDNF-AS,<br>BDNF	BMI (4-7)<br>Hip circumference (10)<br>Obesity (8)<br>Overweight (5, 8)<br>Waist circumference (10)
rs7104764	11:229977	SIRT3	/
rs7138803	12:49853685		BMI (4-8, 23, 25)<br>Hip circumference (10)<br>Obesity (4, 8, 22-24)<br>Overweight (4, 5, 23)<br>Waist circumference (10)
rs7141210	14:100716133		/
rs7215990	17:6131511		/
rs7463166	8:4976268	CSMD1	Height (2)
rs7514705	1:74541036	FPGT-TNNI3K,<br>TNNI3K	BMI (4-7)<br>Hip circumference (10)<br>Overweight (5)<br>Waist circumference (10)
rs7642134	3:86867732		/
rs7647973	3:49473498	DAG1	/
rs7701886	5:154167849		BMI (26)
rs7759938	6:104931079		Height (2, 5, 8, 11, 14-16, 29-31)<br>Pubertal anthropometrics (30)
rs7821178	8:77181601		Height (2)
rs7828501	8:4702559	CSMD1	/
rs7853970	9:84100651		/
rs7865468	9:10274080	PTPRD	/
rs7955374	12:47486366		/
rs8032675	15:67667126	MAP2K5	BMI (5, 6)<br>Obesity (8)
rs8050136	16:53782363	FTO	Adiposity (32)<br>BMI (4-8, 23, 33-36)<br>Body fat percentage (21, 32)<br>Body weight (23)<br>Diabetes mellitus type 2 (6, 34, 37-43)<br>Fasting insulin (44)<br>Hip circumference (10)

SNP	Chr: Position	Gene	Secondary phenotypes
			Obesity (8, 22, 33, 36, 45)<br>Overweight (5, 8, 23)<br>Waist circumference (5, 10, 35, 46)<br>Waist to hip ration (5, 10)
rs852069	20:17141948		/
rs889122	19:9885191	OLFM2	/
rs900400	3:157080986		Birth weight (47, 48)<br>Gestational age (48)<br>New born fat mass (49)<br>Ponderal index (47, 48)<br>Waist hip ratio (10)
rs913588	9:7174673	KDM4C	/
rs929843	16:70011845	PDXDC2P	/
rs9321659	6:99668216		/
rs939317	3:184328011	EIF4G1	/
rs9447700	6:76458369		/
rs9475752	6:56915943	DST	/
rs951366	1:205716224	NUCKS1	/
rs9560113	13:111531001		/
rs9635759	17:51536424		/
rs9647570	5:167943258	TENM2	/
rs9849248	3:88192124		/
rs988913	6:54891510	FAM83B	/

References

1. Staley JR, Blackshaw J, Kamat MA, Ellis S, Surendran P, Sun BB, et al. PhenoScanner: a database of human genotype-phenotype associations. Bioinformatics (Oxford, England). 2016;32(20):3207-9.
2. Wood AR, Esko T, Yang J, Vedantam S, Pers TH, Gustafsson S, et al. Defining the role of common variation in the genomic and biological architecture of adult human height. Nat Genet. 2014;46(11):1173-86.
3. Willer CJ, Speliotes EK, Loos RJ, Li S, Lindgren CM, Heid IM, et al. Six new loci associated with body mass index highlight a neuronal influence on body weight regulation. Nat Genet. 2009;41(1):25-34.
4. Speliotes EK, Willer CJ, Berndt SI, Monda KL, Thorleifsson G, Jackson AU, et al. Association analyses of 249,796 individuals reveal 18 new loci associated with body mass index. Nat Genet. 2010;42(11):937-48.
5. Randall JC, Winkler TW, Kutalik Z, Berndt SI, Jackson AU, Monda KL, et al. Sex-stratified genome-wide association studies including 270,000 individuals show sexual dimorphism in genetic loci for anthropometric traits. PLoS Genet. 2013;9(6):e1003500.
6. Gaulton KJ, Ferreira T, Lee Y, Raimondo A, Magi R, Reschen ME, et al. Genetic fine mapping and genomic annotation defines causal mechanisms at type 2 diabetes susceptibility loci. Nat Genet. 2015;47(12):1415-25.
7. Winkler TW, Justice AE, Graff M, Barata L, Feitosa MF, Chu S, et al. The Influence of Age and Sex on Genetic Associations with Adult Body Size and Shape: A Large-Scale Genome-Wide Interaction Study. PLoS Genet. 2015;11(10):e1005378.
8. Berndt SI, Gustafsson S, Magi R, Ganna A, Wheeler E, Feitosa MF, et al. Genome-wide meta-analysis identifies 11 new loci for anthropometric traits and provides insights into genetic architecture. Nat Genet. 2013;45(5):501-12.

9. Graff M, Ngwa JS, Workalemahu T, Homuth G, Schipf S, Teumer A, et al. Genome-wide analysis of BMI in adolescents and young adults reveals additional insight into the effects of genetic loci over the life course. Hum Mol Genet. 2013;22(17):3597-607.
10. Shungin D, Winkler TW, Croteau-Chonka DC, Ferreira T, Locke AE, Magi R, et al. New genetic loci link adipose and insulin biology to body fat distribution. Nature. 2015;518(7538):187-96.
11. Lango Allen H, Estrada K, Lettre G, Berndt SI, Weedon MN, Rivadeneira F, et al. Hundreds of variants clustered in genomic loci and biological pathways affect human height. Nature. 2010;467(7317):832-8.
12. Stahl EA, Raychaudhuri S, Remmers EF, Xie G, Eyre S, Thomson BP, et al. Genome-wide association study meta-analysis identifies seven new rheumatoid arthritis risk loci. Nat Genet. 2010;42(6):508-14.
13. Lauc G, Huffman JE, Pucic M, Zgaga L, Adamczyk B, Muzinic A, et al. Loci associated with N-glycosylation of human immunoglobulin G show pleiotropy with autoimmune diseases and haematological cancers. PLoS Genet. 2013;9(1):e1003225.
14. He M, Xu M, Zhang B, Liang J, Chen P, Lee JY, et al. Meta-analysis of genome-wide association studies of adult height in East Asians identifies 17 novel loci. Hum Mol Genet. 2015;24(6):1791-800.
15. Soler Artigas M, Loth DW, Wain LV, Gharib SA, Obeidat M, Tang W, et al. Genome-wide association and large-scale follow up identifies 16 new loci influencing lung function. Nat Genet. 2011;43(11):1082-90.
16. Fatemifar G, Hoggart CJ, Paternoster L, Kemp JP, Prokopenko I, Horikoshi M, et al. Genome-wide association study of primary tooth eruption identifies pleiotropic loci associated with height and craniofacial distances. Hum Mol Genet. 2013;22(18):3807-17.
17. Cousminer DL, Stergiakouli E, Berry DJ, Ang W, Groen-Blokhuis MM, Korner A, et al. Genome-wide association study of sexual maturation in males and females highlights a role for body mass and menarche loci in male puberty. Hum Mol Genet. 2014;23(16):4452-64.
18. Arking DE, Pulit SL, Crotti L, van der Harst P, Munroe PB, Koopmann TT, et al. Genetic association study of QT interval highlights role for calcium signaling pathways in myocardial repolarization. Nat Genet. 2014;46(8):826-36.
19. Ganesh SK, Zakai NA, van Rooij FJ, Soranzo N, Smith AV, Nalls MA, et al. Multiple loci influence erythrocyte phenotypes in the CHARGE Consortium. Nat Genet. 2009;41(11):1191-8.
20. Perry JR, Day F, Elks CE, Sulem P, Thompson DJ, Ferreira T, et al. Parent-of-origin-specific allelic associations among 106 genomic loci for age at menarche. Nature. 2014;514(7520):92-7.
21. Lu Y, Day FR, Gustafsson S, Buchkovich ML, Na J, Bataille V, et al. New loci for body fat percentage reveal link between adiposity and cardiometabolic disease risk. Nat Commun. 2016;7:10495.
22. Bradfield JP, Taal HR, Timpson NJ, Scherag A, Lecoeur C, Warrington NM, et al. A genome-wide association meta-analysis identifies new childhood obesity loci. Nat Genet. 2012;44(5):526-31.
23. Thorleifsson G, Walters GB, Gudbjartsson DF, Steinthorsdottir V, Sulem P, Helgadottir A, et al. Genome-wide association yields new sequence variants at seven loci that associate with measures of obesity. Nat Genet. 2009;41(1):18-24.
24. Wheeler E, Huang N, Bochukova EG, Keogh JM, Lindsay S, Garg S, et al. Genome-wide SNP and CNV analysis identifies common and low-frequency variants associated with severe early-onset obesity. Nat Genet. 2013;45(5):513-7.
25. Guo Y, Lanktree MB, Taylor KC, Hakonarson H, Lange LA, Keating BJ, et al. Gene-centric meta-analyses of 108 912 individuals confirm known body mass index loci and reveal three novel signals. Hum Mol Genet. 2013;22(1):184-201.
26. Monda KL, Chen GK, Taylor KC, Palmer C, Edwards TL, Lange LA, et al. A meta-analysis identifies new loci associated with body mass index in individuals of African ancestry. Nat Genet. 2013;45(6):690-6.
27. Okada Y, Wu D, Trynka G, Raj T, Terao C, Ikari K, et al. Genetics of rheumatoid arthritis contributes to biology and drug discovery. Nature. 2014;506(7488):376-81.

28. Jostins L, Ripke S, Weersma RK, Duerr RH, McGovern DP, Hui KY, et al. Host-microbe interactions have shaped the genetic architecture of inflammatory bowel disease. Nature. 2012;491(7422):119-24.
29. Elks CE, Perry JR, Sulem P, Chasman DI, Franceschini N, He C, et al. Thirty new loci for age at menarche identified by a meta-analysis of genome-wide association studies. Nat Genet. 2010;42(12):1077-85.
30. Cousminer DL, Berry DJ, Timpson NJ, Ang W, Thiering E, Byrne EM, et al. Genome-wide association and longitudinal analyses reveal genetic loci linking pubertal height growth, pubertal timing and childhood adiposity. Hum Mol Genet. 2013;22(13):2735-47.
31. Widen E, Ripatti S, Cousminer DL, Surakka I, Lappalainen T, Jarvelin MR, et al. Distinct variants at LIN28B influence growth in height from birth to adulthood. Am J Hum Genet. 2010;86(5):773-82.
32. Kilpelainen TO, Zillikens MC, Stancakova A, Finucane FM, Ried JS, Langenberg C, et al. Genetic variation near IRS1 associates with reduced adiposity and an impaired metabolic profile. Nat Genet. 2011;43(8):753-60.
33. Wan ES, Cho MH, Boutaoui N, Klanderman BJ, Sylvia JS, Ziniti JP, et al. Genome-wide association analysis of body mass in chronic obstructive pulmonary disease. Am J Respir Cell Mol Biol. 2011;45(2):304-10.
34. Timpson NJ, Lindgren CM, Weedon MN, Randall J, Ouwehand WH, Strachan DP, et al. Adiposity-related heterogeneity in patterns of type 2 diabetes susceptibility observed in genome-wide association data. Diabetes. 2009;58(2):505-10.
35. Heard-Costa NL, Zillikens MC, Monda KL, Johansson A, Harris TB, Fu M, et al. NRXN3 is a novel locus for waist circumference: a genome-wide association study from the CHARGE Consortium. PLoS Genet. 2009;5(6):e1000539.
36. Paternoster L, Evans DM, Nohr EA, Holst C, Gaborieau V, Brennan P, et al. Genome-wide population-based association study of extremely overweight young adults--the GOYA study. PloS One. 2011;6(9):e24303.
37. Scott LJ, Mohlke KL, Bonnycastle LL, Willer CJ, Li Y, Duren WL, et al. A genome-wide association study of type 2 diabetes in Finns detects multiple susceptibility variants. Science. 2007;316(5829):1341-5.
38. Zeggini E, Weedon MN, Lindgren CM, Frayling TM, Elliott KS, Lango H, et al. Replication of genome-wide association signals in UK samples reveals risk loci for type 2 diabetes. Science. 2007;316(5829):1336-41.
39. Replication DIG, Meta-analysis C, Asian Genetic Epidemiology Network Type 2 Diabetes C, South Asian Type 2 Diabetes C, Mexican American Type 2 Diabetes C, Type 2 Diabetes Genetic Exploration by Nex-generation sequencing in muylti-Ethnic Samples C, et al. Genome-wide trans-ancestry meta-analysis provides insight into the genetic architecture of type 2 diabetes susceptibility. Nat Genet. 2014;46(3):234-44.
40. Wellcome Trust Case Control C. Genome-wide association study of 14,000 cases of seven common diseases and 3,000 shared controls. Nature. 2007;447(7145):661-78.
41. Lettre G, Palmer CD, Young T, Ejebe KG, Allayee H, Benjamin EJ, et al. Genome-wide association study of coronary heart disease and its risk factors in 8,090 African Americans: the NHLBI CARe Project. PLoS Genet. 2011;7(2):e1001300.
42. Cho YS, Chen CH, Hu C, Long J, Ong RT, Sim X, et al. Meta-analysis of genome-wide association studies identifies eight new loci for type 2 diabetes in east Asians. Nat Genet. 2012;44(1):67-72.
43. Takeuchi F, Serizawa M, Yamamoto K, Fujisawa T, Nakashima E, Ohnaka K, et al. Confirmation of multiple risk Loci and genetic impacts by a genome-wide association study of type 2 diabetes in the Japanese population. Diabetes. 2009;58(7):1690-9.
44. Scott RA, Lagou V, Welch RP, Wheeler E, Montasser ME, Luan J, et al. Large-scale association analyses identify new loci influencing glycemic traits and provide insight into the underlying biological pathways. Nat Genet. 2012;44(9):991-1005.

45. Cotsapas C, Speliotes EK, Hatoum IJ, Greenawalt DM, Dobrin R, Lum PY, et al. Common body mass index-associated variants confer risk of extreme obesity. Hum Mol Genet. 2009;18(18):3502-7.
46. Lindgren CM, Heid IM, Randall JC, Lamina C, Steinthorsdottir V, Qi L, et al. Genome-wide association scan meta-analysis identifies three Loci influencing adiposity and fat distribution. PLoS Genet. 2009;5(6):e1000508.
47. Freathy RM, Mook-Kanamori DO, Sovio U, Prokopenko I, Timpson NJ, Berry DJ, et al. Variants in ADCY5 and near CCNL1 are associated with fetal growth and birth weight. Nat Genet. 2010;42(5):430-5.
48. Horikoshi M, Yaghootkar H, Mook-Kanamori DO, Sovio U, Taal HR, Hennig BJ, et al. New loci associated with birth weight identify genetic links between intrauterine growth and adult height and metabolism. Nat Genet. 2013;45(1):76-82.
49. Urbanek M, Hayes MG, Armstrong LL, Morrison J, Lowe LP, Badon SE, et al. The chromosome 3q25 genomic region is associated with measures of adiposity in newborns in a multi-ethnic genome-wide association study. Hum Mol Genet. 2013;22(17):3583-96.
