# Supplementary material for: Age at menarche and lung function: a Mendelian randomization study
Source: Eur J Epidemiol. 2017 Jun 17;32(8):701–10. doi: 10.1007/s10654-017-0272-9 (PMC5591357; doi:10.1007/s10654-017-0272-9)
Supplement: Supplementary file 3 — Estimates of the SNP-age at menarche association (GX) for all 122 SNPs, from Perry et al. EA: effect allele; EAF: effect allele frequency; R2: proportion of the variance of age at menarche explained by the SNP, calculated as follows: R2 = [2 × EAF × (1–EAF) × β2]/varX, where β is the estimated genetic effect on age at menarche and varX is the variance of age at menarche; F: F statistic, a function of the magnitude and precision of the genetic effect calculated as: F = R2(N − 2)/(1 − R2), where N is the sample size of the SNP-age of menarche association (N = 182, 416); GX: per-allele genetic effect on age at menarche (years); GX SE: standard error of GX; p: p value of GX (PDF 401 kb) [file 10654_2017_272_MOESM3_ESM.pdf]

Supplementary Table 3. Estimates of the SNP-age at menarche association (GX) for all 122 SNPs from Perry et al. (Nature 2014; 514:92-7). *EA* : effect allele; *EAF* : effect allele frequency; R^2 : proportion of the variance of age at menarche explained by the SNP, calculated as follows: $R^2=[2\times EAF\times(1-EAF)\times GX^2]/varX$, where varX is the variance of age at menarche; *F* : F statistic, a function of the magnitude and precision of the genetic effect calculated as: $F=R^2(N-2)/(1-R^2)$, where N is the sample size for GX (N=182,416) (Palmer et al. Stat Methods Med Res 2012; 21:223-42); *GX* : per-allele genetic effect on age at menarche (years); *GX SE* : standard error of GX; *p* : p value of GX.

SNP	Gene	EA	EAF	R^2	F	GX	GX SE	p
rs10144321	*DLK1*	a	0.75	0.0002	42	0.04	0.006	9.0E-15
rs1038903	*PCDH7*	t	0.73	0.0002	44	0.04	0.006	2.0E-11
rs10423674	*CRTC1*	a	0.34	0.0003	50	0.04	0.005	9.2E-12
rs10453225	*TMEM38B*	g	0.68	0.0014	248	0.09	0.005	5.8E-66
rs10739221	*TMEM38B*	c	0.77	0.0009	159	0.08	0.006	3.9E-41
rs10789181	*LEPR*	a	0.39	0.0002	30	0.03	0.005	3.5E-08
rs1079866	*INHBA*	g	0.15	0.0005	88	0.07	0.007	9.3E-24
rs10816359	*TMEM38B*	t	0.86	0.0001	27	0.04	0.008	1.6E-08
rs10895140	*TRPC6, PGR*	g	0.66	0.0003	50	0.04	0.005	6.7E-14
rs10938397	*GNPDA2*	a	0.57	0.0003	55	0.04	0.005	4.0E-13
rs10980854	*ZNF483 / OR2K2*	a	0.06	0.0002	28	0.06	0.011	1.3E-08
rs10980921	*ZNF483 / OR2K2*	c	0.09	0.0005	93	0.09	0.009	1.7E-23
rs11022756	*ARNTL, PTH*	a	0.29	0.0004	72	0.05	0.006	7.4E-20
rs11165924	*DPYD*	a	0.69	0.0001	27	0.03	0.006	2.2E-09
rs11215400	*CADM1*	c	0.27	0.0002	44	0.04	0.006	6.8E-11
rs1129700	*KCTD13, TBX6*	t	0.44	0.0002	31	0.03	0.005	2.3E-09
rs11578152	*OLFM3*	g	0.44	0.0002	31	0.03	0.005	4.5E-08
rs11715566	*IGSF11*	t	0.5	0.0005	88	0.05	0.005	2.4E-27
rs11767400	*CADPS2*	a	0.3	0.0003	47	0.04	0.006	4.1E-11
rs11792861	*TMEM245*	a	0.7	0.0003	47	0.04	0.005	1.7E-11
rs12148769	*MKRN3, MAGEL2*	g	0.9	0.0002	32	0.05	0.008	5.2E-11
rs12446632	*GPRC5B*	a	0.13	0.0001	25	0.04	0.007	1.3E-08
rs12472911	*LRP1B*	c	0.2	0.0002	36	0.04	0.006	6.7E-10
rs1254337	*SIX6*	t	0.31	0.0003	48	0.04	0.005	2.1E-16
rs12571664	*SEC23IP*	t	0.79	0.0002	37	0.04	0.006	3.3E-10
rs12607903	*DLGAP1*	c	0.3	0.0003	47	0.04	0.005	5.4E-11
rs12915845	*DET1*	c	0.58	0.0002	31	0.03	0.005	2.7E-12
rs13053505	*NPTXR, CBX7*	g	0.8	0.0002	36	0.04	0.007	3.0E-08
rs13067731	*IL20RB*	t	0.16	0.0002	30	0.04	0.007	1.0E-09
rs13135934	*SMARCAD1*	c	0.4	0.0002	30	0.03	0.005	1.1E-10
rs13179411	*PHF15, TCF7*	t	0.17	0.0004	71	0.06	0.007	3.4E-20
rs13196561	*SIM1, MCHR2*	c	0.78	0.0002	39	0.04	0.006	8.4E-12
rs1324913	*KLF12*	g	0.65	0.0002	29	0.03	0.005	3.1E-10
rs1364063	*COG4, NFAT5*	c	0.43	0.0005	86	0.05	0.005	6.2E-21
rs1400974	*SATB2*	a	0.64	0.0004	81	0.05	0.005	8.3E-20
rs1461503	*BSX*	c	0.57	0.0005	86	0.05	0.005	2.7E-26
rs1469039	*KCNK9*	a	0.19	0.0003	54	0.05	0.007	3.5E-12

SNP	Gene	EA	EAF	R^2	F	GX	GX SE	p
rs1532331	*ZNF131, GHR*	g	0.32	0.0002	27	0.03	0.005	3.5E-09
rs16860328	*TRA2B, IGF2BP2*	g	0.42	0.0003	55	0.04	0.005	1.4E-16
rs16896742	*HLA-A*	g	0.38	0.0003	53	0.04	0.006	3.2E-10
rs16918254	*NPBWR1*	a	0.92	0.0001	26	0.05	0.009	1.4E-08
rs16918636	*FSHB*	t	0.79	0.0001	21	0.03	0.006	3.2E-08
rs17086188	*PCSK1*	a	0.94	0.0002	39	0.07	0.013	3.6E-08
rs17171818	*KDM3B, BRD8*	c	0.77	0.0002	40	0.04	0.006	8.9E-14
rs17233066	*SATB2*	c	0.93	0.0004	74	0.09	0.014	6.1E-11
rs17236969	*NR4A2*	t	0.14	0.0002	42	0.05	0.008	2.6E-09
rs17266097	*SATB2*	t	0.42	0.0003	55	0.04	0.005	3.3E-18
rs1874984	*ADARB2*	c	0.47	0.0003	56	0.04	0.005	1.9E-12
rs1915146	*CTBP2*	g	0.4	0.0002	30	0.03	0.005	3.7E-08
rs1958560	*FUT8*	a	0.59	0.0002	31	0.03	0.005	3.7E-08
rs2063730	*GAB2, THRSP*	c	0.18	0.0003	52	0.05	0.007	2.3E-12
rs2137289	*SKOR2*	a	0.59	0.0005	85	0.05	0.005	8.2E-20
rs2153127	*LIN28B*	t	0.52	0.0012	224	0.08	0.005	5.5E-59
rs2274465	*KDM4A, PTPRF*	c	0.66	0.0002	28	0.03	0.005	1.7E-09
rs239198	*SIM1, ASCC3*	t	0.46	0.0002	31	0.03	0.005	2.5E-08
rs244293	*STXBP4*	g	0.6	0.0002	30	0.03	0.005	4.2E-11
rs246185	*MKL2*	c	0.33	0.0003	50	0.04	0.006	6.8E-16
rs2479724	*BYSL, FRS3*	t	0.45	0.0002	31	0.03	0.005	1.2E-12
rs251130	*STARD4*	g	0.73	0.0002	44	0.04	0.006	2.8E-10
rs2600959	*ACAD11*	a	0.34	0.0003	50	0.04	0.005	4.1E-11
rs268067	*BCL11A*	a	0.8	0.0002	36	0.04	0.006	3.3E-08
rs2687729	*EEFSEC*	g	0.27	0.0002	44	0.04	0.006	1.0E-10
rs2688325	*CSMD1*	t	0.29	0.0001	26	0.03	0.006	2.1E-09
rs2836950	*BRWD1*	c	0.64	0.0002	29	0.03	0.005	6.2E-11
rs2947411	*TMEM18*	a	0.17	0.0004	71	0.06	0.007	1.8E-19
rs3101336	*NEGR1*	t	0.4	0.0003	54	0.04	0.005	5.2E-13
rs3733631	*TACR3*	c	0.15	0.0002	45	0.05	0.007	4.8E-13
rs3743266	*RORA*	t	0.68	0.0003	49	0.04	0.005	2.4E-13
rs4369815	*NR4A2*	t	0.93	0.0002	33	0.06	0.01	1.5E-10
rs466639	*RXRG*	c	0.87	0.0006	102	0.08	0.007	2.4E-24
rs4756059	*PHF21A*	t	0.92	0.0003	51	0.07	0.01	4.5E-13
rs4840086	*SIM1, MCHR2*	a	0.58	0.0003	55	0.04	0.005	9.2E-14
rs4875053	*SCRIB, PARP10*	g	0.44	0.0002	31	0.03	0.006	1.3E-08
rs4895808	*CENPW, NCOA7*	c	0.54	0.0002	31	0.03	0.005	4.8E-13
rs4929947	*TRIM66*	g	0.36	0.0003	52	0.04	0.005	2.6E-12
rs543874	*SEC16B*	a	0.8	0.0003	56	0.05	0.006	1.4E-15
rs6009583	*C22orf34*	c	0.74	0.0001	24	0.03	0.006	4.6E-08
rs6427782	*NR5A2*	a	0.51	0.0002	32	0.03	0.005	4.6E-08
rs652260	*EVI5L, RETN*	t	0.54	0.0002	31	0.03	0.005	9.9E-09
rs6555855	*SLIT3*	g	0.23	0.0002	40	0.04	0.006	2.4E-09
rs6563739	*COG6*	g	0.34	0.0002	28	0.03	0.005	2.3E-11
rs6747380	*CCDC85A*	a	0.17	0.0005	97	0.07	0.007	5.6E-28
rs6758290	*GPR45*	t	0.5	0.0003	56	0.04	0.005	6.6E-13

SNP	Gene	EA	EAF	R^2	F	GX	GX SE	p
rs6762477	*WDR6, UBA7*	g	0.44	0.0003	55	0.04	0.006	7.8E-12
rs6770162	*THRB*	a	0.51	0.0003	56	0.04	0.005	1.5E-12
rs6933660	*ESR1*	c	0.69	0.0001	27	0.03	0.005	1.3E-09
rs6938574	*PTPRK*	t	0.16	0.0002	30	0.04	0.007	2.4E-09
rs6964833	*GTF2I*	t	0.75	0.0002	42	0.04	0.006	5.3E-12
rs7037266	*KDM4C*	a	0.37	0.0002	29	0.03	0.005	4.7E-09
rs7103411	*BDNF, LGR4*	c	0.21	0.0002	37	0.04	0.006	2.6E-11
rs7104764	*SIRT3*	g	0.25	0.0001	24	0.03	0.006	3.7E-08
rs7138803	*BCDIN3D*	g	0.62	0.0003	53	0.04	0.005	1.7E-12
rs7141210	*DLK1*	t	0.34	0.0002	28	0.03	0.005	5.8E-09
rs7215990	*WSCD1 , ALOX15B*	g	0.76	0.0002	41	0.04	0.006	1.9E-08
rs7463166	*CSMD1*	a	0.63	0.0002	29	0.03	0.005	1.3E-08
rs7514705	*TNNI3K, TYW3*	c	0.56	0.0003	55	0.04	0.005	1.8E-16
rs7642134	*POU1F1 (PIT1)*	g	0.61	0.0003	53	0.04	0.005	3.0E-16
rs7647973	*WDR6, UBA7*	a	0.26	0.0004	68	0.05	0.006	1.3E-16
rs7701886	*GALNT10*	a	0.58	0.0002	31	0.03	0.005	4.5E-08
rs7759938	*LIN28B*	c	0.32	0.0024	441	0.12	0.005	7.8E-110
rs7821178	*PEX2*	c	0.65	0.0003	51	0.04	0.005	7.3E-17
rs7828501	*CSMD1*	g	0.45	0.0003	56	0.04	0.005	1.2E-13
rs7853970	*RMI1, NTRK2*	t	0.47	0.0002	31	0.03	0.005	2.3E-09
rs7865468	*PTPRD*	a	0.7	0.0001	27	0.03	0.005	1.3E-07
rs7955374	*VDR*	t	0.13	0.0001	25	0.04	0.008	9.5E-09
rs8032675	*MAP2K5*	t	0.4	0.0003	54	0.04	0.005	2.1E-13
rs8050136	*FTO*	c	0.6	0.0003	54	0.04	0.005	1.7E-17
rs852069	*PCSK2*	g	0.64	0.0003	52	0.04	0.005	1.2E-13
rs889122	*OLFM2, RDH8*	g	0.72	0.0002	45	0.04	0.006	1.6E-13
rs900400	*LEKR1, CCNL1*	t	0.61	0.0002	30	0.03	0.005	2.3E-11
rs913588	*KDM4C*	g	0.49	0.0002	32	0.03	0.005	5.8E-11
rs929843	*COG4, WWP2*	a	0.23	0.0002	40	0.04	0.006	1.2E-11
rs9321659	*SIM1, MCHR2*	a	0.13	0.0003	57	0.06	0.008	2.5E-16
rs939317	*EIF4G1*	g	0.74	0.0002	43	0.04	0.006	3.0E-12
rs9447700	*IMPG1*	c	0.69	0.0001	27	0.03	0.005	5.6E-09
rs9475752	*DST, BEND6*	c	0.81	0.0002	35	0.04	0.006	8.3E-12
rs951366	*NUCKS1, RAB7L1*	t	0.6	0.0002	30	0.03	0.005	1.7E-08
rs9560113	*TEX29*	g	0.28	0.0004	71	0.05	0.006	2.1E-17
rs9635759	*CA10*	a	0.32	0.0004	76	0.05	0.005	1.7E-24
rs9647570	*TENM2*	g	0.14	0.0002	42	0.05	0.007	1.4E-11
rs9849248	*ZNF654, HTR1F*	c	0.15	0.0002	29	0.04	0.007	1.9E-08
rs988913	*FAM83B, HCRTR2*	c	0.66	0.0003	50	0.04	0.005	1.4E-12
