# Supplementary material for: Age at menarche and lung function: a Mendelian randomization study
Source: Eur J Epidemiol. 2017 Jun 17;32(8):701–10. doi: 10.1007/s10654-017-0272-9 (PMC5591357; doi:10.1007/s10654-017-0272-9)
Supplement: Supplementary file 4 — Estimates of the SNP-lung function association (GY) for all 122 SNPs, for adult women (ECRHS, NFBC 1966 and UK Biobank studies) and adolescent girls (ALSPAC and NFBC 1986 studies). For one SNP, rs10423674, data were missing from NFBC 1986. EA: effect allele; GY: per-allele genetic effect on FVC (ml) or FEV1/FVC (%); GY SE: standard error of GY (PDF 407 kb) [file 10654_2017_272_MOESM4_ESM.pdf]

Supplementary Table 4. Estimates of the SNP-lung function association (GY) for all 122 SNPs, for adult women (ECRHS, NFBC 1966 and UK Biobank studies) and adolescent girls (ALSPAC and NFBC 1986 studies). For one SNP, rs10423674, data were missing from NFBC 1986. *EA* : effect allele; *GY* : per-allele genetic effect on FVC (ml) or FEV1/FVC (%); *GY SE* : standard error of GY.

		FVC				FEV1/FVC			
		Adults (n=46,944)		Adolescents (n=3,025)		Adults (n=46,944)		Adolescents (n=3,025)	
SNP	EA	GY	GY SE	GY	GY SE	GY	GY SE	GY	GY SE
rs10144321	a	-0.62	5.72	-10.17	12.54	-0.05	0.17	0.10	0.18
rs1038903	t	3.72	5.57	-7.67	12.36	0.00	0.17	-0.05	0.18
rs10423674	a	5.30	5.18	-4.33	18.69	0.02	0.14	0.02	0.25
rs10453225	g	17.78	5.24	-8.68	11.66	0.16	0.15	0.11	0.18
rs10739221	c	17.29	5.85	-2.32	12.81	-0.02	0.16	0.08	0.20
rs10789181	a	1.62	5.05	5.68	11.30	-0.18	0.15	0.07	0.17
rs1079866	g	-1.06	7.06	-14.15	15.09	-0.02	0.18	0.27	0.23
rs10816359	t	7.09	6.64	-2.08	15.66	0.41	0.20	0.09	0.24
rs10895140	g	-8.47	5.17	-14.26	11.45	0.08	0.15	0.12	0.18
rs10938397	a	8.15	4.98	-2.50	10.95	-0.02	0.14	-0.03	0.17
rs10980854	a	-4.22	10.49	-53.31	21.53	0.29	0.26	0.00	0.33
rs10980921	c	-8.95	9.00	-22.14	20.71	0.15	0.26	0.21	0.29
rs11022756	a	2.86	5.42	-18.07	11.89	-0.06	0.16	0.04	0.18
rs11165924	a	10.13	5.28	15.53	12.45	-0.26	0.16	0.44	0.18
rs11215400	c	4.12	5.60	17.64	12.36	0.11	0.16	0.00	0.18
rs1129700	t	-0.09	4.94	-21.66	11.13	0.17	0.15	0.18	0.17
rs11578152	g	5.14	4.92	1.51	11.00	0.11	0.14	-0.12	0.16
rs11715566	t	7.07	4.90	11.47	11.16	0.10	0.15	0.06	0.16
rs11767400	a	-11.28	5.41	-6.67	12.30	0.06	0.16	0.29	0.18
rs11792861	a	3.87	5.38	8.42	12.15	0.01	0.15	-0.07	0.18
rs12148769	g	8.29	8.23	34.88	18.95	-0.40	0.24	-0.33	0.28
rs12446632	a	-7.74	7.07	-19.77	16.09	-0.02	0.22	-0.48	0.26
rs12472911	c	-6.70	5.82	-5.65	13.31	0.00	0.16	-0.19	0.19
rs1254337	t	-0.96	5.37	8.48	12.97	-0.13	0.16	-0.23	0.19
rs12571664	t	-0.04	6.25	-8.87	13.79	0.33	0.17	0.06	0.21
rs12607903	c	-0.97	5.47	-11.10	12.15	0.20	0.15	0.05	0.18
rs12915845	c	-1.60	4.97	-11.57	11.28	-0.12	0.14	0.33	0.17
rs13053505	g	-3.05	6.52	11.24	13.49	0.05	0.17	0.22	0.20
rs13067731	t	-10.09	6.67	17.94	15.47	0.11	0.19	0.00	0.23
rs13135934	c	-5.26	5.02	-9.66	11.37	0.16	0.15	-0.19	0.17
rs13179411	t	8.87	6.67	8.77	14.16	-0.04	0.17	-0.15	0.22
rs13196561	c	10.18	6.06	-9.41	13.62	-0.13	0.17	-0.22	0.20
rs1324913	g	3.58	5.20	2.75	11.84	-0.24	0.15	0.07	0.17
rs1364063	c	16.90	5.00	-20.39	11.33	-0.07	0.14	0.00	0.17
rs1400974	a	-5.91	5.17	10.24	12.02	0.17	0.16	0.25	0.18
rs1461503	c	-4.75	4.93	-8.21	11.01	-0.01	0.14	0.22	0.17
rs1469039	a	-4.80	6.38	8.36	13.01	-0.05	0.16	0.21	0.19

SNP	EA	FVC				FEV1/FVC			
		Adults (n=46,944)		Adolescents (n=3,025)		Adults (n=46,944)		Adolescents (n=3,025)	
		GY	GY SE	GY	GY SE	GY	GY SE	GY	GY SE
rs1532331	g	4.83	5.32	10.74	11.29	0.28	0.14	-0.37	0.17
rs16860328	g	1.82	4.98	10.96	11.39	-0.03	0.14	0.13	0.17
rs16896742	g	8.87	5.54	14.62	11.42	-0.34	0.28	0.00	0.17
rs16918254	a	10.41	9.45	37.50	22.75	0.01	0.27	0.03	0.33
rs16918636	t	1.80	6.15	-28.55	15.03	0.08	0.20	0.56	0.23
rs17086188	a	19.98	11.25	-15.05	17.80	-0.16	0.21	0.39	0.31
rs17171818	c	-9.02	6.09	-7.28	13.31	0.02	0.17	-0.02	0.20
rs17233066	c	-9.57	10.34	31.47	32.19	-0.06	0.37	-0.13	0.50
rs17236969	t	-3.38	6.99	-5.38	18.60	-0.12	0.26	0.13	0.26
rs17266097	t	12.75	5.02	3.51	11.18	-0.15	0.14	0.25	0.17
rs1874984	c	-1.41	4.93	21.65	11.36	0.03	0.14	-0.15	0.17
rs1915146	g	-5.35	4.96	6.69	11.54	-0.11	0.14	-0.09	0.18
rs1958560	a	2.96	5.00	-1.77	11.22	0.28	0.15	0.14	0.17
rs2063730	c	7.43	6.22	4.58	13.93	0.07	0.17	-0.35	0.21
rs2137289	a	11.51	5.09	11.96	11.56	0.10	0.14	-0.13	0.17
rs2153127	t	-18.45	4.94	-12.54	11.36	-0.23	0.15	0.24	0.17
rs2274465	c	-0.57	5.18	-12.39	11.49	-0.06	0.15	0.15	0.17
rs239198	t	0.68	4.94	-6.00	11.04	-0.08	0.14	-0.09	0.17
rs244293	g	-2.36	5.10	-18.35	11.57	-0.04	0.14	0.18	0.18
rs246185	c	8.18	5.27	16.40	11.65	0.18	0.14	-0.30	0.18
rs2479724	t	-4.08	4.89	-5.24	11.09	-0.08	0.14	-0.08	0.16
rs251130	g	-2.26	5.51	-23.95	12.05	0.12	0.16	0.07	0.18
rs2600959	a	4.91	5.18	-14.45	11.98	0.01	0.14	-0.24	0.17
rs268067	a	6.35	6.27	5.75	13.95	-0.28	0.17	0.44	0.21
rs2687729	g	1.80	5.53	-23.99	11.97	-0.16	0.15	-0.07	0.18
rs2688325	t	4.54	5.37	-22.93	12.09	0.21	0.15	0.17	0.18
rs2836950	c	11.25	5.10	17.46	11.14	0.08	0.14	-0.20	0.16
rs2947411	a	2.34	6.55	17.54	14.72	0.13	0.18	-0.03	0.22
rs3101336	t	-0.81	5.02	-8.27	11.32	0.28	0.15	0.24	0.17
rs3733631	c	-13.27	6.76	10.88	14.65	0.10	0.19	-0.25	0.24
rs3743266	t	-1.43	5.33	-12.30	12.91	0.09	0.18	0.18	0.19
rs4369815	t	7.52	10.32	-8.02	25.98	-0.39	0.34	-0.77	0.35
rs466639	c	9.25	7.42	21.10	16.05	0.06	0.20	-0.48	0.24
rs4756059	t	-6.99	9.68	16.08	21.60	-0.02	0.28	-0.01	0.31
rs4840086	a	-0.56	4.95	-2.24	11.16	0.15	0.14	-0.01	0.17
rs4875053	g	2.38	5.06	6.92	10.94	0.13	0.14	0.30	0.17
rs4895808	c	-16.69	4.94	-26.94	11.00	0.24	0.14	0.21	0.17
rs4929947	g	10.41	5.14	6.10	11.67	-0.05	0.15	0.02	0.18
rs543874	a	6.26	6.05	3.00	14.18	-0.53	0.17	0.34	0.20
rs6009583	c	-2.32	5.64	-19.36	12.93	0.06	0.17	-0.03	0.19
rs6427782	a	6.63	4.93	-1.44	11.06	-0.03	0.14	0.05	0.17
rs652260	t	-4.10	4.93	-13.13	11.42	-0.26	0.14	0.11	0.17
rs6555855	g	3.31	5.97	-1.34	12.56	0.17	0.17	0.15	0.19
rs6563739	g	3.03	5.13	20.23	11.97	0.00	0.16	-0.25	0.17

SNP	EA	FVC				FEV1/FVC			
		Adults (n=46,944)		Adolescents (n=3,025)		Adults (n=46,944)		Adolescents (n=3,025)	
		GY	GY SE	GY	GY SE	GY	GY SE	GY	GY SE
rs6747380	a	0.88	6.38	-8.43	13.30	0.05	0.17	-0.36	0.21
rs6758290	t	1.10	4.95	5.71	11.11	0.08	0.15	-0.12	0.16
rs6762477	g	-18.09	4.93	16.12	11.47	0.00	0.14	-0.02	0.17
rs6770162	a	-1.23	4.93	0.94	11.56	0.10	0.14	-0.01	0.17
rs6933660	c	10.57	5.26	-2.88	12.15	-0.03	0.15	0.06	0.18
rs6938574	t	9.83	6.81	-18.70	17.30	0.21	0.23	-0.07	0.24
rs6964833	t	-9.80	5.65	-17.23	13.40	-0.26	0.16	0.12	0.20
rs7037266	a	7.75	5.11	-0.26	11.13	-0.04	0.14	0.09	0.17
rs7103411	c	4.33	6.06	-5.54	14.70	-0.36	0.18	0.37	0.20
rs7104764	g	2.03	5.72	27.96	12.73	-0.03	0.17	-0.06	0.19
rs7138803	g	1.80	5.08	-9.26	11.81	-0.30	0.15	-0.13	0.17
rs7141210	t	-7.60	5.18	-0.29	11.82	0.14	0.15	-0.06	0.17
rs7215990	g	-2.35	5.83	-2.56	13.53	0.09	0.17	0.04	0.20
rs7463166	a	-2.19	5.13	-12.45	11.56	-0.21	0.14	0.13	0.17
rs7514705	c	-2.19	5.01	-18.10	11.02	0.16	0.14	0.31	0.16
rs7642134	g	9.46	5.05	2.28	11.64	0.14	0.15	-0.14	0.17
rs7647973	a	-5.39	5.67	-21.71	13.13	-0.24	0.18	-0.14	0.20
rs7701886	a	5.22	4.97	1.18	11.64	0.08	0.14	0.20	0.17
rs7759938	c	-13.92	5.27	-13.49	12.22	-0.04	0.15	0.27	0.18
rs7821178	c	4.85	5.24	-0.12	12.41	-0.03	0.16	0.06	0.19
rs7828501	g	10.80	4.97	4.19	11.41	-0.02	0.14	-0.11	0.17
rs7853970	t	-0.06	5.02	-23.97	11.19	0.00	0.15	0.17	0.17
rs7865468	a	-0.68	5.39	-8.60	11.89	-0.04	0.15	-0.07	0.18
rs7955374	t	18.03	7.49	10.70	17.04	-0.22	0.21	-0.15	0.26
rs8032675	t	-5.97	5.04	-17.24	11.48	0.13	0.15	0.05	0.17
rs8050136	c	9.03	5.00	-2.98	11.41	0.02	0.14	-0.01	0.17
rs852069	g	12.21	5.09	-2.16	11.33	-0.06	0.15	-0.07	0.17
rs889122	g	-4.13	5.47	3.98	11.98	0.10	0.16	0.22	0.18
rs900400	t	-13.15	5.25	0.62	11.35	0.05	0.15	-0.16	0.17
rs913588	g	-6.74	4.90	0.00	10.89	-0.03	0.15	-0.08	0.16
rs929843	a	14.71	6.04	19.83	14.69	-0.29	0.18	-0.05	0.22
rs9321659	a	-14.86	7.42	-7.47	17.93	0.33	0.23	-0.01	0.25
rs939317	g	-2.18	5.61	-17.43	11.89	-0.06	0.15	0.30	0.19
rs9447700	c	-3.28	5.29	-12.25	11.77	-0.06	0.14	-0.20	0.17
rs9475752	c	-5.91	6.14	-6.85	14.32	0.27	0.17	0.25	0.20
rs951366	t	-8.34	4.61	11.64	11.36	-0.24	0.15	-0.34	0.17
rs9560113	g	8.94	5.48	11.02	12.28	-0.27	0.16	0.10	0.18
rs9635759	a	5.68	5.37	-2.92	11.79	0.10	0.16	-0.22	0.18
rs9647570	g	8.33	7.02	-2.92	14.71	-0.14	0.19	-0.20	0.22
rs9849248	c	8.63	6.79	-20.78	14.36	-0.16	0.18	-0.07	0.22
rs988913	c	8.91	5.10	-5.39	11.33	0.03	0.14	0.05	0.17
