# Supplementary material for: Age at menarche and lung function: a Mendelian randomization study
Source: Eur J Epidemiol. 2017 Jun 17;32(8):701–10. doi: 10.1007/s10654-017-0272-9 (PMC5591357; doi:10.1007/s10654-017-0272-9)
Supplement: Supplementary file 5 — Estimates of the causal effect of age at menarche on lung function for all 122 SNPs, for adult women (ECRHS, NFBC 1966 and UK Biobank studies) and adolescent girls (ALSPAC and NFBC 1986 studies). EA: effect allele; Beta: estimate of the effect of one year increase in age at menarche on FVC (ml) or FEV1/FVC (%); Beta SE: standard error of beta (PDF 499 kb) [file 10654_2017_272_MOESM5_ESM.pdf]

Supplementary Table 5. Estimates of the causal effect of age at menarche on lung function for all 122 SNPs, for adults (ECRHS, NFBC 1966, UK Biobank) and adolescents (ALSPAC, NFBC 1986). *EA* : effect allele; *Beta* : estimate of the effect of one year increase in age at menarche on FVC (ml) or FEV_1/FVC (%); *Beta SE* : standard error of beta.

		FVC				FEV1/FVC			
		Adults (n=46,944)		Adolescents (n=3,025)		Adults (n=46,944)		Adolescents (n=3,025)	
**SNP**	**EA**	**Beta**	**Beta SE**	**Beta**	**Beta SE**	**Beta**	**Beta SE**	**Beta**	**Beta SE**
rs10144321	a	-15.5	143.0	-254.3	315.8	-1.3	4.3	2.5	4.5
rs1038903	t	93.0	139.9	-191.8	310.3	0.5	3.5	-1.3	4.5
rs10423674	a	132.5	130.6	-108.3	467.4	1.8	1.7	0.5	6.3
rs10453225	g	197.6	59.2	-96.4	129.7	-0.3	2.0	1.2	2.0
rs10739221	c	216.1	74.9	-29.0	160.1	-6.0	5.1	1.0	2.5
rs10789181	a	54.0	168.6	189.3	378.0	-0.3	2.6	2.3	5.7
rs1079866	g	-15.1	100.9	-202.1	216.5	10.3	5.4	3.9	3.3
rs10816359	t	177.3	169.7	-52.0	391.6	2.0	3.8	2.3	6.0
rs10895140	g	-211.8	131.9	-356.5	289.7	-0.5	3.5	3.0	4.5
rs10938397	a	203.8	127.1	-62.5	273.9	4.8	4.4	-0.8	4.3
rs10980854	a	-70.3	175.3	-888.5	394.1	1.7	2.9	2.3	3.2
rs10980921	c	-99.4	100.5	-246.0	231.4	-1.2	3.2	0.8	3.6
rs11022756	a	57.2	108.6	-361.4	241.7	-8.7	5.6	14.7	6.7
rs11165924	a	337.7	188.5	517.7	427.7	2.8	4.0	6.0	5.8
rs11215400	c	103.0	140.8	441.0	316.0	5.7	5.1	-4.0	5.4
rs1129700	t	-3.0	164.7	-722.0	390.0	3.7	4.7	1.2	3.2
rs11578152	g	171.3	166.5	50.3	366.8	2.0	3.0	7.3	4.6
rs11715566	t	141.4	99.0	229.4	224.4	1.5	4.0	-1.8	4.5
rs11767400	a	-282.0	141.7	-166.8	308.5	0.3	3.8	-6.6	5.7
rs11792861	a	96.8	135.0	210.5	304.9	-8.0	5.0	-12.0	6.8
rs12148769	g	165.8	166.7	697.6	395.1	-0.5	5.5	-4.8	4.8
rs12446632	a	-193.5	180.0	-494.3	411.4	-3.3	4.0	-5.8	4.8
rs12472911	c	-167.5	147.7	-141.3	333.4	8.3	4.4	1.5	5.3
rs1254337	t	-24.0	134.3	212.0	325.3	5.0	3.8	1.3	4.5
rs12571664	t	-1.0	156.2	-221.8	346.3	-4.0	4.7	11.0	6.0
rs12607903	c	-24.3	136.8	-277.5	305.7	1.3	4.3	5.5	5.1
rs12915845	c	-53.3	165.9	-385.7	381.4	2.8	4.8	-6.3	5.8
rs13053505	g	-76.3	163.5	281.0	340.8	5.3	5.1	-2.5	3.7
rs13067731	t	-252.3	172.5	448.5	394.6	-0.7	2.8	-5.5	5.1
rs13135934	c	-175.3	169.9	-322.0	382.8	-3.3	4.3	2.3	5.7
rs13179411	t	147.8	112.5	146.2	236.6	-8.0	5.2	5.0	3.6
rs13196561	c	254.5	156.2	-235.3	342.3	-1.4	2.8	4.4	3.4
rs1324913	g	119.3	174.5	91.7	395.0	3.4	3.2	4.2	3.8
rs1364063	c	338.0	105.6	-407.8	230.2	-0.2	2.8	-12.3	6.0
rs1400974	a	-118.2	104.1	204.8	241.3	-1.0	3.2	3.3	4.3
rs1461503	c	-95.0	99.1	-164.2	220.8	9.3	4.9	0.6	6.6
rs1469039	a	-96.0	128.3	167.2	261.2	-0.8	3.5	18.7	8.5
rs1532331	g	161.0	179.3	358.0	381.0	-8.5	7.1	5.6	4.5

		FVC				FEV1/FVC			
		Adults (n=46,944)		Adolescents (n=3,025)		Adults (n=46,944)		Adolescents (n=3,025)	
**SNP**	**EA**	**Beta**	**Beta SE**	**Beta**	**Beta SE**	**Beta**	**Beta SE**	**Beta**	**Beta SE**
rs16860328	g	45.5	124.6	274.0	286.8	0.2	5.4	-0.5	5.0
rs16896742	g	221.8	142.4	365.5	290.7	2.7	6.7	-1.4	5.6
rs16918254	a	208.2	192.7	750.0	474.6	-2.3	3.0	2.6	5.2
rs16918636	t	60.0	205.3	-951.7	535.9	0.5	4.3	6.3	4.3
rs17086188	a	285.4	169.2	-215.0	257.4	-0.7	4.1	-3.8	4.3
rs17171818	c	-225.5	156.0	-182.0	333.9	-2.4	5.2	-3.0	6.0
rs17233066	c	-106.3	116.1	349.7	361.8	-3.8	3.5	4.7	5.7
rs17236969	t	-67.6	140.2	-107.6	372.4	0.8	3.5	-7.0	4.3
rs17266097	t	318.8	131.7	87.8	279.7	-3.7	4.7	-2.6	3.4
rs1874984	c	-35.3	123.3	541.3	291.9	9.3	5.2	3.0	2.1
rs1915146	g	-178.3	168.0	223.0	386.5	1.4	3.4	5.0	5.7
rs1958560	a	98.7	167.5	-59.0	374.1	2.0	2.8	-3.0	5.7
rs2063730	c	148.6	126.1	91.6	278.9	-2.9	1.9	6.0	6.1
rs2137289	a	230.2	104.4	239.2	232.4	-2.0	5.0	-7.5	4.6
rs2153127	t	-230.6	63.4	-156.8	142.3	-2.7	4.7	-2.7	5.4
rs2274465	c	-19.0	172.7	-413.0	389.1	-1.3	4.7	1.8	4.5
rs239198	t	22.7	164.7	-200.0	369.5	4.5	3.6	-6.0	4.3
rs244293	g	-78.7	170.5	-611.7	398.9	-2.7	4.7	11.0	5.5
rs246185	c	204.5	135.3	410.0	297.7	3.0	4.0	-1.8	4.5
rs2479724	t	-136.0	164.6	-174.7	370.8	0.3	3.5	5.7	6.1
rs251130	g	-56.5	138.0	-598.8	314.3	-7.0	4.4	-6.7	5.4
rs2600959	a	122.8	130.4	-361.3	302.9	-4.0	3.8	-0.5	3.7
rs268067	a	158.8	158.5	143.8	349.4	7.0	5.2	6.0	4.3
rs2687729	g	45.0	138.4	-599.8	312.5	2.7	4.7	-5.0	4.9
rs2688325	t	151.3	181.5	-764.3	431.0	2.2	3.0	4.5	4.8
rs2836950	c	375.0	181.1	582.0	383.8	7.0	3.9	-12.8	6.2
rs2947411	a	39.0	109.3	292.3	247.7	2.0	3.8	-6.0	3.0
rs3101336	t	-20.3	125.5	-206.8	284.2	2.3	4.5	-0.1	4.4
rs3733631	c	-265.4	140.2	217.6	294.6	-6.5	5.8	-0.3	4.3
rs3743266	t	-35.8	133.3	-307.5	325.0	0.8	2.5	10.0	6.0
rs4369815	t	125.3	173.3	-133.7	433.6	-0.3	4.0	7.0	5.8
rs466639	c	115.6	93.3	263.8	201.9	3.8	3.5	0.5	4.5
rs4756059	t	-99.9	139.0	229.7	310.3	4.3	4.7	6.8	4.1
rs4840086	a	-14.0	123.8	-56.0	279.1	8.0	4.9	-1.0	6.3
rs4875053	g	79.3	169.4	230.7	367.6	-1.3	3.8	1.7	5.7
rs4895808	c	-556.3	189.0	-898.0	396.0	-10.6	3.6	3.7	5.7
rs4929947	g	260.3	132.6	152.5	292.4	2.0	5.7	3.8	4.8
rs543874	a	125.2	121.9	60.0	283.7	-1.0	4.7	-8.3	5.8
rs6009583	c	-77.3	188.6	-645.3	449.9	-8.7	4.9	-5.1	3.0
rs6427782	a	221.0	168.4	-48.0	368.7	4.3	4.3	-3.0	4.0
rs652260	t	-136.7	165.9	-437.7	387.6	0.7	2.4	-0.5	4.3
rs6555855	g	82.8	149.8	-33.5	314.0	2.0	3.8	-0.3	4.3
rs6563739	g	101.0	171.8	674.3	414.5	2.5	3.5	2.0	6.0

		FVC				FEV1/FVC			
		Adults (n=46,944)		Adolescents (n=3,025)		Adults (n=46,944)		Adolescents (n=3,025)	
**SNP**	**EA**	**Beta**	**Beta SE**	**Beta**	**Beta SE**	**Beta**	**Beta SE**	**Beta**	**Beta SE**
rs6747380	a	12.6	91.2	-120.4	190.4	-1.0	5.0	-1.8	6.0
rs6758290	t	27.5	123.8	142.8	278.3	5.3	5.8	3.0	5.0
rs6762477	g	-452.3	140.7	403.0	293.0	-6.5	4.1	3.0	5.7
rs6770162	a	-30.8	123.3	23.5	289.0	-1.3	4.7	9.3	5.2
rs6933660	c	352.3	184.9	-96.0	405.3	-9.0	4.7	-2.0	6.3
rs6938574	t	245.8	175.6	-467.5	440.2	-1.0	5.7	-3.3	4.3
rs6964833	t	-245.0	146.0	-430.8	341.2	-7.5	3.9	-2.0	5.7
rs7037266	a	258.3	175.7	-8.7	371.0	4.7	5.1	1.0	5.0
rs7103411	c	108.3	152.4	-138.5	368.1	2.3	4.3	4.3	5.7
rs7104764	g	67.7	191.1	932.0	463.5	-7.0	4.8	7.8	4.1
rs7138803	g	45.0	127.1	-231.5	296.7	4.0	3.5	-3.5	4.3
rs7141210	t	-253.3	177.8	-9.7	394.0	3.5	3.8	-2.8	4.0
rs7215990	g	-58.8	146.0	-64.0	338.4	-4.8	3.6	6.7	5.8
rs7463166	a	-73.0	171.4	-415.0	391.5	2.7	4.7	2.3	1.5
rs7514705	c	-54.8	125.4	-452.5	281.2	-0.3	1.3	1.5	4.8
rs7642134	g	236.5	129.7	57.0	291.1	-0.8	4.0	-2.8	4.3
rs7647973	a	-107.8	114.1	-434.2	267.7	-0.5	3.5	5.7	5.7
rs7701886	a	174.0	168.2	39.3	388.0	-1.3	5.0	-2.3	6.0
rs7759938	c	-116.0	44.2	-112.4	101.9	-5.5	5.4	-3.8	6.5
rs7821178	c	121.3	131.9	-3.0	310.2	3.3	3.8	1.3	4.3
rs7828501	g	270.0	128.8	104.8	285.5	0.5	3.5	-0.3	4.3
rs7853970	t	-2.0	167.3	-799.0	396.1	-1.5	3.8	-1.8	4.3
rs7865468	a	-22.7	179.7	-286.7	399.2	2.5	4.0	5.5	4.6
rs7955374	t	450.8	207.8	267.5	429.3	1.7	5.0	-5.3	5.7
rs8032675	t	-149.3	127.4	-431.0	292.0	-1.0	5.0	-2.7	5.4
rs8050136	c	225.8	128.1	-74.5	285.4	-7.3	4.6	-1.3	5.5
rs852069	g	305.3	132.8	-54.0	283.3	5.5	3.9	-0.2	4.2
rs889122	g	-103.3	137.6	99.5	299.9	-1.5	3.8	7.5	4.9
rs900400	t	-438.3	189.6	20.7	378.3	-2.0	4.7	-6.7	5.8
rs913588	g	-224.7	167.6	495.8	374.7	6.8	4.4	6.3	5.1
rs929843	a	367.8	160.8	-124.5	299.3	-8.0	5.2	-11.3	6.0
rs9321659	a	-247.7	128.0	-435.8	304.3	-5.4	3.3	2.0	3.6
rs939317	g	-54.5	140.5	-408.3	398.2	2.0	3.2	-4.4	3.6
rs9447700	c	-109.3	177.3	-171.3	358.9	-2.8	3.8	-4.0	4.4
rs9475752	c	-147.8	155.1	388.0	384.1	-4.0	4.6	-1.8	5.5
rs951366	t	-278.0	160.5	220.4	247.0	0.8	3.5	1.3	4.3
rs9560113	g	178.8	111.7	-58.4	235.9	-4.0	4.6	1.9	3.7
rs9635759	a	113.6	108.0	-58.4	294.3	0.8	3.5	-4.3	3.6
rs9647570	g	166.6	142.3	-519.5	370.3	-2.8	3.9	-4.0	4.4
rs9849248	c	215.8	173.9	-134.8	283.7	-4.0	4.7	-1.9	5.6
rs988913	c	222.8	130.5	-134.8	283.8	0.7	3.5	1.2	4.1
