# Supplementary material for: Age at menarche and lung function: a Mendelian randomization study
Source: Eur J Epidemiol. 2017 Jun 17;32(8):701–10. doi: 10.1007/s10654-017-0272-9 (PMC5591357; doi:10.1007/s10654-017-0272-9)
Supplement: Supplementary file 6 — Characteristics of the study populations included for the SNP-lung function associations in adult men and adolescent boys. Values reported are mean (standard deviation) (PDF 312 kb) [file 10654_2017_272_MOESM6_ESM.pdf]

Supplementary Table 6

Supplementary Table 6. Characteristics of the studies included for the SNP-lung function associations in adult men and adolescent boys. Values reported are mean (standard deviation)

**Study**	**Study design**	**Sample size** (N)	**Age at spirometry** (years)	**FVC** (ml)	**FEV1** (ml)	**FEV_1/FVC** (%)
**Adult men**						
ECRHS II	Multicentre cohort	1,063	42.8 (7.2)	5,118 (869)	4,014 (752)	79 (7.6)
NFBC 1966	Birth cohort	2,450	31 (0)	5,493 (751)	4,550 (622)	83 (6.4)
UK Biobank	Multicentre cross-sectional	37,174	57.2 (8.0)	4,272 (918)	3169 (800)	74 (7.8)
**Adolescent men**						
ALSPAC	Birth cohort	1,717	15.5 (0.3)	4,229 (850)	3,750 (757)	89 (7.6)
NFBC 1986	Birth cohort	1,704	16 (0)	4,917 (729)	4,230 (623)	86 (7.4)
